# A biomechanical study of gait initiation in Down syndrome

**DOI:** 10.1186/s12883-019-1288-4

**Published:** 2019-04-15

**Authors:** Carolina Corsi, Veronica Cimolin, Paolo Capodaglio, Claudia Condoluci, Manuela Galli

**Affiliations:** 1 Department of Electronics, Information and Bioengineering, Politecnico di Milano, Piazza Leonardo da Vinci 32, 20133 Milan, Italy; 2 Orthopaedic Rehabilitation Unit and Clinical Lab for Gait Analysis and Posture, Ospedale San Giuseppe, Istituto Auxologico Italiano, IRCCS, Via Cadorna 90, I-28824, Piancavallo, VB Italy; 3 IRCCS San Raffaele Pisana, via della Pisana 235, 00163 Rome, Italy

**Keywords:** Down syndrome, Gait initiation, Biomechanics

## Abstract

**Background:**

Gait Initiation (GI) is a functional task that challenges the balance control requiring weight shift and a transition from standing to walking. Individuals with Down Syndrome (DS) walk with low velocity, prolonged stance and shorter steps beside an increased support base. However, no studies performed GI analysis on this population. The aim of this study is to quantitatively characterize the GI task in subjects with DS compared with a typically developed control group.

**Methods:**

Seventeen individuals with DS (17 to 40 years) and 19 healthy subjects (17 to 40 years) were enrolled in the study. Data were acquired using an optoelectronic motion capture system and force plates in order to measure the displacement and velocity of Center of Mass (CoM) and the trajectory of Center Of Pressure (CoP). All participants were asked to stand barefoot on the first force platform and received a verbal cue to begin walking for 6 gait initiation trials (three starting with each foot). The CoP duration, velocity, length and excursion were calculated during the anticipatory postural adjustments phases (APAs) and the locomotor (LOC) phase. For the analysis of the CoM, its displacements in antero-posterior (AP) and medio-lateral (ML) during the APAs and LOC phases. Statistical analysis was conducted to compare the two groups.

**Results:**

Regarding CoP measures, when compared to control group, individuals with DS presented higher durations, lower velocities, longer lengths during the second APA and total phases, and shorter lengths during the first APA and LOC phases. The group with DS also presented longer CoP excursion during the second APA, whereas a shorter excursion was present during the first APA and LOC phases. The AP excursion in CoM is reduced in the participants with DS.

**Conclusions:**

Our results could be useful in the rehabilitation of individuals with DS as they suggest to reinforce exercise programs to improve balance in AP and ML directions, which is demonstrated to be impaired in these subjects.

## Background

Down Syndrome (DS) is a genetic condition caused by the presence of all or part of a third CoPy of chromosome 21, and for this reason, is also called as “Trisomy 21” [1]. There is a large individual variability in phenotypic and clinical manifestations in individuals with DS, however, DS is considered one of the most common causes of mental impairment [2]. Children with DS present delay in acquisition of main motor milestones, especially in the acquisition of trunk control, erect posture and walking [2, 3], which can be attributed to hypotonia, a deficit in equilibrium mechanisms and ligament laxity, often present in an individual with DS [4]. Actually, previous studies hypothesized that the presence of cerebellar hypoplasia in this population is the cause of muscular hypotonia and of difficulties in motor coordination [5, 6].

The peculiar motor deficit in children with DS is the slowness of movements, which persists even in adulthood. Although children with DS learn to walk, reach and take objects, their movements lack coordination, precision and are less effective than people with typical development. In addition, these individuals have “clumsy” movements and little control over programming in multiple movements involving different joints [7].

Studies addressing the postural control in subjects with DS found that these subjects use a different balance control with respect to healthy subjects. Galli et al. [8] analyzed ground reaction force and center of pressure track with a time domain approach and a frequency domain approach of subjects with DS in an upright position; they found instabilities in both lateral and anterior-posterior directions, indicating that during the upright position, compensatory strategies are required and activated providing greater control and stability in this population. Different strategies also occur during walking: the most obvious feature is slowness, with a prolonged stance and shorter steps. Individuals with DS tend to increase the support base by widening the lower limbs. Additionally, they show an increased hip flexion as compared to typical developing subjects, widening them even more horizontally (more intrinsic) to ensure forward progression [9, 10].

Gait initiation [GI] is the transient period between the quiet standing posture and steady state walking, with several anticipatory anterior-posterior and lateral movements [11–13]. GI is typically associated with anticipatory postural adjustments [APAs] and occurs prior to gross segmental movement and stability-boundary changes of the first step [14]. GI cycle starts with weight evenly distributed between the limbs with the center of pressure [CoP] located between the feet during quiet standing, and ends at toe-off of the stance limb.

Traditionally, movement analysis of individuals with DS has prevalently focused on the biomechanical evaluation and description of a limited set of movements [7, 15–18].

In particular, most quantitative studies are related to gross motor abilities of everyday life, such as walking and posture [8, 19–22]. In patients with DS the movements related to transitions from a static posture (standing) to dynamic task could be difficult. For this reason the analysis of GI could be of particular interest when the study of functional limitations as well as motor control related to this pathological condition is required.

The GI analysis in people with DS to the best of our knowledge is not yet conducted. Therefore, the hypothesis of this study is that the slowness, longer reaction times, instability, and patterns of muscular co-contractions typical of individuals it DS might delay movement initiation. Thus, the aim of this study is to quantitatively characterize the GI in subjects with DS compared with a typically developed control group of healthy subjects, using parameters derived from the Center of Pressure (COP) and Center of Mass (CoM) tracks.

## Methods

### Subjects

The sample of the present study consisted of two groups. One composed of subjects with DS (DSG) with of 17 individuals: 9 males and 8 females (median age (quartile range): 36.6 (6.6) years; median height (quartile range): 1.51 (0.08) m; median BMI (quartile range): 37.2 (5.8) kg/m^2^). The other group consisted in a control group (CG) with 19 healthy subjects: 9 males and 10 females (median age (quartile range): 33.9 (11.2) years; median height (quartile range): 1.72 (0.08) m; median BMI (quartile range): 21.4 (1.3) kg/m^2^).

The DSG was selected among individuals with DS assessed at the IRCSS “San Raffaele Pisana” Movement Analysis Laboratory in Rome. For this study, selection criteria were: absence of visual and auditory disorders (assessed with traditional vision and audiological test), absence of congenital cardiac abnormalities, low to medium intelligence quotient (IQ mean: 56.2; range: 44–80) and ability to walk and maintain the erect position without the help of auxiliaries or medical personnel. The healthy subjects were recruited among the hospital staff of San Giuseppe Hospital, Istituto Auxologico Italiano, in Piancavallo [Italy]. Inclusion criteria for the CG were no cardiovascular, neurological or musculoskeletal disorders. They had normal flexibility and muscle strength and no obvious gait abnormalities. All participants were able to walk independently without aids.

Ethical approval of the study was granted by the IRCCS San Raffaele Pisana Ethics Committee (Protocol identification number: SPOL – 17/17 – 6/2017). All the participants were properly informed about aims of the research, testing procedures and personal data treatment. All procedures performed in the study were in accordance with the ethical standards of the institutional and national research committee and with the 1964 Helsinki declaration and its later amendments or comparable ethical standards. All participants were volunteers and gave written informed consent which was confirmed by parents if necessary.

### Equipment

The equipment used for the acquisition and the processing of all data in the two motion analysis laboratories (San Raffaele Pisana - Rome and San Giuseppe Hospital – Piancavallo), includes an optoelectronic motion capture system and two force plates. In particular, at the IRCSS “San Raffaele Pisana” Movement Analysis Laboratory in Rome, there are 12 cameras (ELITE 2002, BTS Bioengineering, Milan, IT); while in the San Giuseppe Hospital, Istituto Auxologico Italiano, in Piancavallo, there are 6 cameras (VICON T40, UK). Although different systems have been used, the data acquired are comparable in accordance with the literature [9]. Regarding the force plates, both labs had two force platforms (KISTLER, CH) that were used for the acquisition of CoP trajectory.

### Experimental set-up

All GI tests were performed in the motion analysis laboratories. Before the acquisition of the data, passive markers were placed on the body of the subjects according to the Davis protocol [23].

The marker positioned at the sacrum was considered as representative in a simplified approach of CoM [24] and its coordinates was used to consider displacement and velocity of the CoM. In order to measure the CoP trajectory, data of interest were acquired using two force platforms. Subjects were instructed to stay on both legs barefoot on the first force platform in a relaxed posture. Acquisition of kinetic data by force platforms was triggered just prior to the participants receiving a verbal cue to begin walking approximately 3 s before starting the task. After the cue, the participants started walking passing through the second platform at a self-selected speed. All the requests were standardized: 3 trials starting with the left foot and 3 trials starting with the right foot.

### Data analysis

#### CoP analysis

**Fig. 1:**
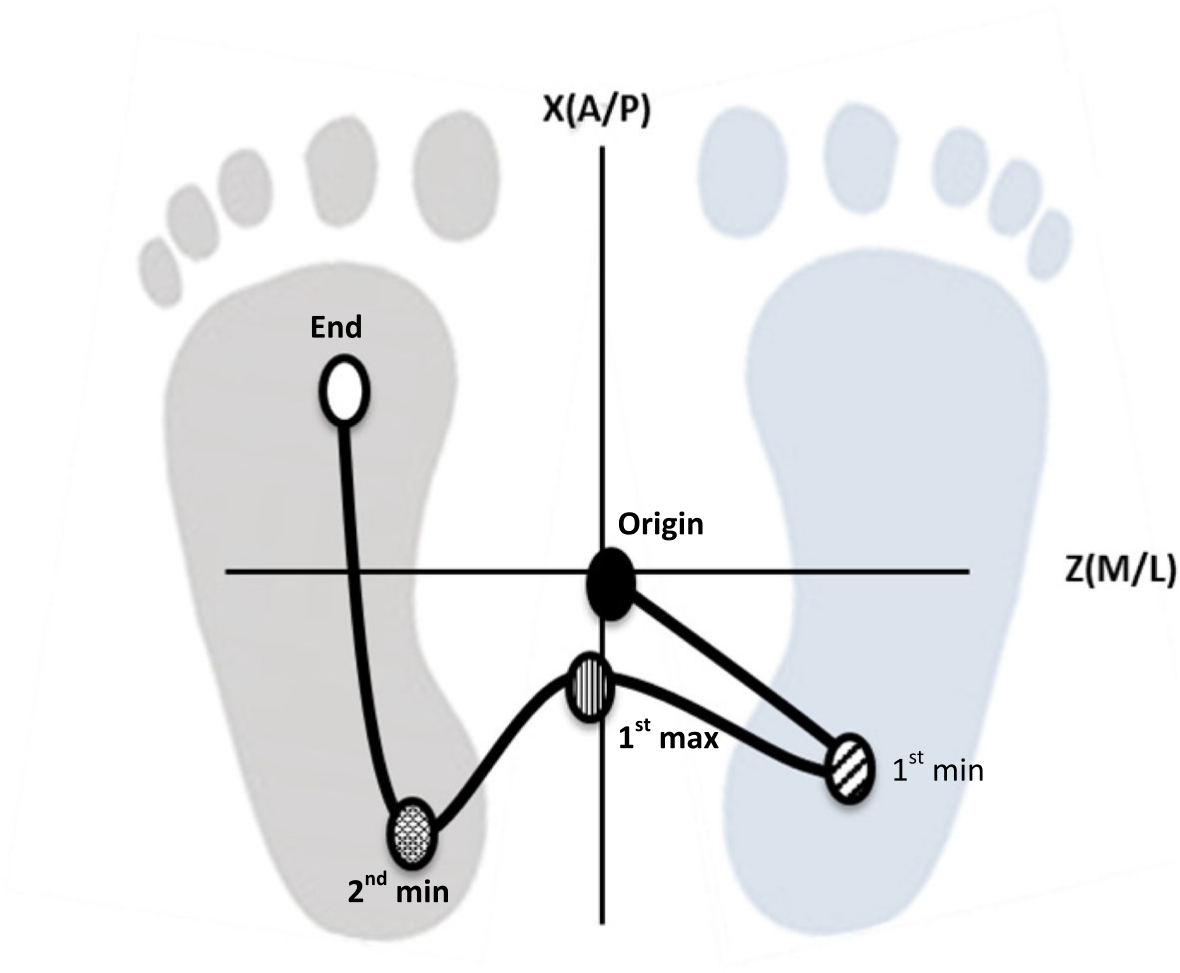
Five points considered in the analysis.  = Origin,  = 1st min;  =1st max  = 2nd min  = End

The analysis of CoP was conducted considering specific parameters on CoP trajectory according to previous papers dedicated to GI study [12, 25–27]. The CoP processing focused on the APAs and the locomotor phase [LOC]. The raw CoP data sampled at a frequency of 1 kHz and low-pass-filtered at 10 Hz was analyzed using a protocol developed using Smart Analyzer software (version: 1.10.451.0; BTS, Italy). For each acquisition, five points were manually identified, according to literature [25, 26] (Fig. 1):

1. Origin (initial CoP position);
2. First minimum (1st min): the minimum posterior position of the CoP on the leg in the swing side;
3. First maximum (1st max): the maximum anterior position during the CoP transition from the leg in the swing to the leg in stance;
4. Second minimum [2nd min]: the minimum posterior position of the CoP on the leg in the stance side;
5. End [Final CoP position].

**Fig. 2:**
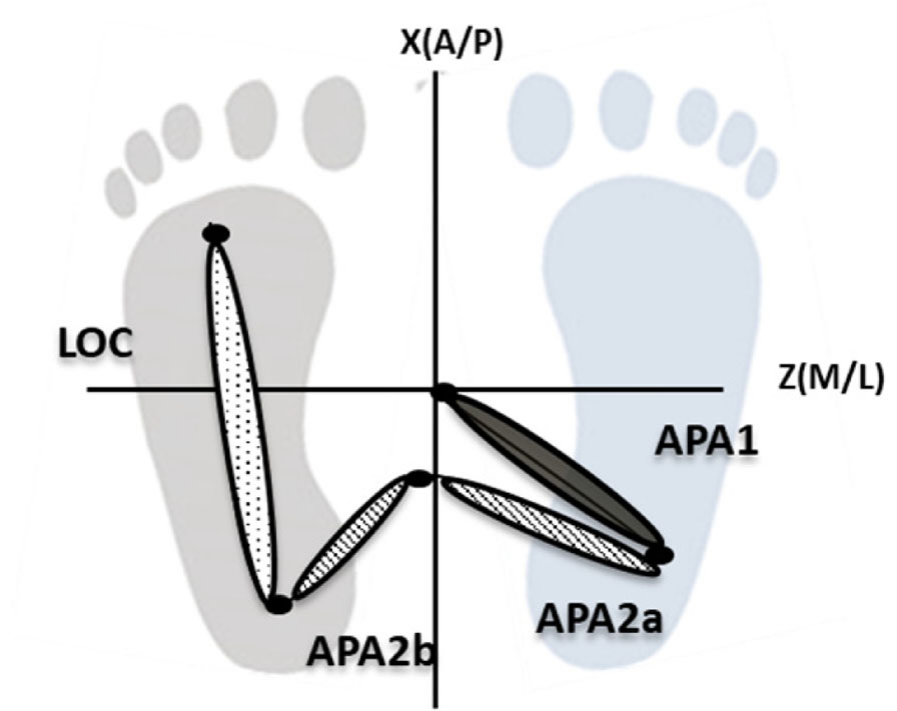
Phases of the GI analysis

For the timing analysis, we divided the task in two phases [12, 25–27] (Fig. 2):

1. Postural phase (computed between a standing position and the start of the task) is divided into two sub-phases (APA1 and APA2 phase):
  APA1, between the origin and the first minimum, representing the translation of the CoP in lateral and posterior directions together toward the swing foot heel.
  APA2, representing the lateral CoP shift toward the stance foot. APA2 was further divided into two additional sub-phases: APA2a (between the first minimum and the first maximum) and APA2b (between the first maximum and the second minimum).
2. Locomotor phase (LOC phase), the phase between the second minimum and the end of the CoP trajectory.

According to the phases subdivision described before, the following parameters have been calculated [25, 26]:

- Track duration [s] during each phase (dAPA1, dAPA2a, dAPA2b, dLOC) and the total duration (dTot);
- Track velocity [m/s] in the anterior-posterior [x] and in medio-lateral directions [z] (vAPA1[x,z], vAPA2a[x,z], vAPA2b[x,z],vLOC [x,z]);
- Track lengths [m] (lAPA1, lAPA2a, lAPA2b, lLOC);
- Track excursion [m] in both the anterior-posterior [x] and in medio lateral directions [z] (eAPA1[x,z], eAPA2a[x,z], eAPA2b[x,z], eLOC [x,z]).

Track length and excursion parameters were normalized respect to the height of the subjects to better appreciate the differences between the groups.

#### CoM analysis

For the analysis of the CoM, 2 phases (previously described and defined based on the CoP) were considered:

1. the first phase = APA1 + APA2 phases
2. the second phase, equal to the generation of a propulsive moment (LOC phase) for the initiation of the gait [28].

**Fig. 3:**
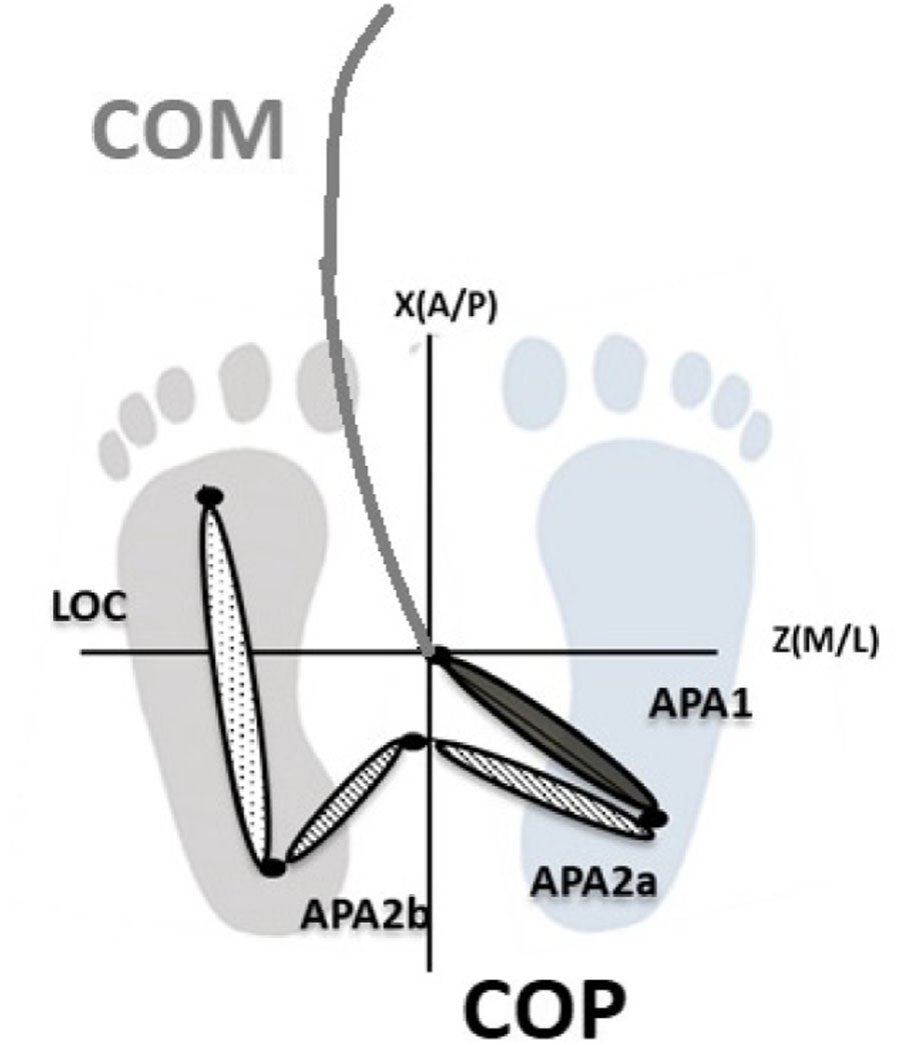
CoM and CoP pattern. After unloading of the right limb the CoP under the stance moves forward under the control of the plantar flexors. During the single support time, the CoM now accelerates forward and away from the stance limb

For these 2 phases, the COM displacements in AP and ML were computed (Fig. 3).

CoM displacement was normalized by the height of the subjects for both AP and ML directions, allowing comparisons between groups. The assessment of CoM versus CoP trajectories (Fig. 3) allows to show [13] that CoP moves posteriorly and towards the swing limb during the release phase. Inverted pendulum theory [13] predicts that this shift could cause the CoM to accelerate in the opposite direction and this is shown in Fig. 3: the CoM trajectories is forward and towards the stance limb. According to literature [13], the CoP posterior movement is due to the decrease in plantar flexion activity. After the release phase, a rapid unload phase occurs. This leads to a rapid shift across to the stance limb. The line joining the CoP and to the COM represents the acceleration vector and after the unload phase this vector is directed forwards and away from the stance foot and forwards towards the future position of the swing foot [13].

### Statistical analysis

Data from a pilot experiment with four subjects with DS and four healthy subjects were used to sample size calculation. The amount of total duration (dTOT) of the task was considered as primary endpoint. A total of 30 subjects (at least 15 per group) was defined as necessary to this study with an 80% power. By convenience, some additional participants were considered in the two groups. All parameters were computed bilaterally for each participant and Kolmogorov-Smirnov tests were used to verify if the parameters were normally distributed. As the parameters were not normally distributed, the median and quartile range values of all indexes were calculated for each group (DSG and CG). Finally, the Mann-Whitney U test was used for comparing data between groups. Level of significance was set at *p* < 0.05. Statistical analysis was conducted using Matlab® software.

## Results

BMI and height were significantly different between the two groups while not age. Preliminary screening of the acquired data were performed to examine possible influences of age, IQ, sex and BMI on CoP and CoM parameters using a Generalized Linear Model; no significant results were obtained.

### CoP analysis

Values of the parameters analyzed for the Control Group (CG) and DS Group (DSG) are summarized in Table 1.

**Table 1:** Values of the median (interquartile range) for calculated parameters for CG and DSG

	Parameters	CG	DSG
APAs and LOC duration (s)	dAPA1	0.21 (0.1)	0.41 (0.02)*
	dAPA2a	0.13 (0.07)	0.30 (0.2)*
	dAPA2b	0.1 (0.05)	0.21 (0.2)*
	dLOC	0.5 (0.07)	0.6 (0.2)*
	dTOT	1.07 (0.2)	1.59 (0.8)*
APAs and LOC velocity (m/s)	vAPA1	0.20 (0.1)	0.09 (0.08)*
	vAPA2a	0.44 (0.2)	0.29 (0.2)*
	vAPA2b	0.43 (0.2)	0.28 (0.2)*
	vLOC	0.31 (0.09)	0.18 (0.08)*
	vAPAs	0.31 (0.09)	0.19 (0.08)*
	vAPA1 AP	0.15 (0.1)	0.03 (0.03)*
	vAPA2a AP	0.14 (0.09)	0.05 (0.07)*
	vAPA2b AP	0.17 (0.1)	0.05 (0.07)*
	vLOC AP	0.2 (0.09)	0.16 (0.07)*
	vAPA1 ML	0.07 (0.07)	0.06 (0.07)*
	vAPA2a ML	0.38 (0.2)	0.23 (0.02)*
	vAPA2b ML	0.39 (0.2)	0.26 (0.02)*
	vLOC ML	0.01 (0.02)	0.021 (0.02)*
APAs and LOC lengt	lAPA1	0.04 (0.1)	0.02 (0.02)*
	lAPA2a	0.03 (0.01)	0.05 (0.02)*
	lAPA2b	0.02 (0.02)	0.04 (0.03)*
	lLOC	0.09 (0.01)	0.07 (0.03)*
	lTOT	0.19 (0.03)	0.2 (0.09)*
APAs and LOC track excursion	eAPA1 AP	0.02 (0.01)	0.01 (0.01)*
	eAPA2a AP	0.01 (0.01)	0.01 (0.01)
	eAPA2b AP	0.01 (0.01)	0.01 (0.01)
	eLOC AP	0.08 (0.01)	0.06 (0.02)*
	eAPA1 ML	0.01 (0.01)	0.01 (0.01)
	eAPA2a ML	0.03 (0.01)	0.04 (0.003)*
	eAPA2b ML	0.02 (0.01)	0.03 (0.02)*
	eLOC ML	0.01 (0.01)	0.01 (0.01)

Legend: *DSG* Down Syndrome Group, *CG* Control Group, *APA* Anticipatory Postural Adjustments, *LOC* locomotor, *d* duration, *v* velocity, *l* length, *e* excursion. * = *p* - value < 0.05

Compared to CG, DSG presented higher durations of all phases, lower velocities in all phases (even for AP and ML directions), longer lengths during APA2a, APA2b and total phases, while it had shorter lengths during APA1 and LOC phases. In terms of excursion parameters, the DSG displayed longer excursion than the CG during APA2a ML and APA2b ML phases, while during APA1 AP, LOC AP and LOC ML phase the DSG presented a shorter excursion than CG.

### CoM analysis

Values of COM parameters analyzed for the control group (CG) and Down syndrome group (DSG) are summarized in Table 2.

**Table 2:** Values of the median (interquartile range) for displacement of COM in ML and AP directions for CG and DSG

Parameters	Phase	Displacement	CG	DSG
CoM Excursion	1	ML	0.006 (0.002)	0.01 (0.008)
		AP	0.01 (0.003)	0.007 (0.04)*
	2	ML	0.01 (0.007)	0.021 (0.013)
		AP	0.117 (0.014)	0.93 (0.03)*

Legend: *CG* Control Group, *DSG* Down Syndrome Group, *CoM* Center Of Mass, *ML* Medio-Lateral, *AP* Antero-Posterior. * = *p* - value < 0.05

Compared to CG, DSG did not differ their ML displacement in both phases related to the APA movement and in the phase related to the anterior advancement of CoP. Otherwise, on AP displacement, DSG presented shorter displacement during CoM phase.

The A/P excursion in CoM is reduced in the DSG, while the ML excursion of the CoP is increased. This data show that the anticipatory adjustments done on the CoP are less effective in the DSG as they have a less CoM movement forward.

## Discussion

This study aims to quantitatively analyze the execution of a motor task, such as that of GI, which involves several motor aspects in individuals with DS. The evaluation of this task in subjects with DS is absent in the literature, which mostly focuses on the evaluation of walking and posture.

In our study, individuals with DS were evaluated using a quantitative method for the characterization of the GI. In this context, it was possible to identify parameters that could best identify the differences between DSG and CG. The first part of this paper focuses only on the CoP analysis and in particular on the APAs and LOC phases. Our results demonstrated that DSG exhibited longer durations than CG during all phases. These results are in line with the presence of motor disturbances common in individuals with DS, which translate in a delay in starting the GI task as compared to controls. In particular, the higher duration could be related to some features of individuals with DS like reduced height, ligamentous laxity, hypotonia and short foot, which may alter the initiation of the task. In addition, significant results were obtained with regard to velocity. For DSG, a reduced and statistically different CoP velocity in AP and in ML direction characterized all of the phases. These results could be connected to the movement slowness of individuals with DS [19] in a continuous balancing effort that could possibly reduce the velocity in anterior-posterior direction. In addition, subjects with DS, show high body masses, which could be related to the higher displacements in the ML direction of the CoM.

In terms of excursion parameters, the higher ML excursion during APA2a and APA2b is similar to studies of GI in obese population [25]. In these studies, the main characteristic is represented by the higher excursion in ML direction, mainly in APA2a and APA2b [25]. The higher body mass of people with DS could lead to reduced propulsion and reduced muscular strength in the lower limbs and it could bring alterations in terms of excursion, especially in ML direction in comparison to the healthy subjects. However, future studies should investigate if this difference during GI is secondary to the syndrome itself or to the body mass.

The second part of this study includes also the CoM analysis. The aim was to investigate CoM changes over time. Both groups presented similar CoP and CoM ML displacements in phase 1 and 2. In terms of the CoP displacement in ML direction, each group significantly increased the CoP displacements in the ML direction so to deliver similar displacements in phase 2. Some researchers [28] suggested that a smaller CoP-ML displacement could be connected to difficulties in GI related to a scarce balance ability.

Regarding the AP displacement, DSG presented shorter displacement during CoP phase 1 and longer displacement on CoP phase 2 when compared to CG. These results imply that DSG needed to adjust the displacements in order to reach a critical amount of displacement to transition to phase 2; on the other hand, CG could generate sufficient CoP-AP displacement for GI in phase 1, confirming literature [28]. This result demonstrates the presence of a predominantly backward strategy during gait initiation. As for CoM, both groups demonstrated greater CoM displacements in phase 2, due to forward movement. However, DSG presented shorter displacement of CoM during phase 1 and 2, which results in small length of steps of this population that is described in the literature. The CoP and CoM trajectory show a decoupling movement during gait initiation. The Cop and CoM are characterized by a synergic movement: while the CoP moves laterally considering the foot in contact, the ML component of CoM moves also forward and towards. Both these CoP and CoM movements are important for lateral stability during GI.

In terms of velocities, DSG showed the lowest CoP velocities in all directions and all phases, except for CoP-ML in phase 2, where no differences were present between groups. This consideration confirms the results obtained in the first part: healthy subjects demonstrate greater CoP- ML velocity, while subjects with DS show a lower velocity during the gait initiation. It is known from previous studies [13, 30] that persons with alterations and deficit in balance have reduced CoP velocity. Slowness of movement, hypotonia and deficit in equilibrium in DS could contribute to the reduced CoP velocity found in the present study.

Some of these results are in agreement with GI performance of obese individuals [25], which showed long APAs length and duration associated to abnormal CoP velocity, if compared with normal-weight individuals. However the results obtained in patients with DS could be related not only to the excessive body mass but also to the syndrome itself.

Our results could be useful in rehabilitation of individuals with DS as they suggest to reinforce exercise programs to improve balance in AP and ML directions, which is demonstrated to be impaired in these subjects [8]. In addition, weight management and tailored strengthening and balance exercises may increase stability and reducing risk of fall in this population. Adipose tissue accumulation and body mass increases can be in fact a major factor contributing to the occurrence of falls, which explains why persons with high body mass appear to be at greater risk than normal-weight subjects [29]. Then, as efficient lower limb muscles are the key to independent mobility, the strength improvement of the flexor and extensor muscles is highly correlated with the capacity to execute daily tasks safely and maintain balance [31].

The limitations of this study were the followings: firstly, only data related to CoP and CoM trajectory were investigated while no evaluations of lower limb joints kinematics and kinetics were conducted. Secondly, the wide age range, resulting in limited strength of the statistical findings. It could be interesting to integrate our analysis including data about the degree of muscular hypotonia, weakness and ligament laxity, not available for this analysis. In addition, as overweight is a distinctive feature in DS, their pattern should have been more rigorously compared with obese instead of normal-weight individuals, in order to identify features related to high body mass and those related to the syndrome itself. On the other hand, the main object of our investigation was to objectively quantify the GI performance respect to normality in DS patients. However, it is important to underline that, despite these limitations, this represents the first study focusing on GI performance in DS subjects.

## Conclusion

Individuals with DS presented higher duration and lower velocity of CoP displacement during all phases of GI, a higher ML excursion during the shift toward the stance foot phase. These findings suggest a difficulty in postural control during GI. This difficult may lead to shorter displacement of CoM during phase 1 and 2 and to reduced step length in this population.

## Abbreviations

AP: Anterior-posterior
APA: Anticipatory postural adjustment
CG: Control group
CoM: Center of Mass
CoP: Center of pressure
DS: Down Syndrome
DSG: Down Syndrome Group
GI: Gait initiation
LOC: Locomotor phase
ML: Medio-lateral
SMA: Supplementary motor area
